# Optogenetically controlled RAF to characterize BRAF and CRAF protein kinase inhibitors

**DOI:** 10.1038/srep23713

**Published:** 2016-03-30

**Authors:** Claire V. Chatelle, Désirée Hövermann, Anne Müller, Hanna J. Wagner, Wilfried Weber, Gerald Radziwill

**Affiliations:** 1 BIOSS - Centre for Biological Signalling Studies, University of Freiburg, Schänzlestr. 18, 79104 Freiburg, Germany; 2 SGBM - Spemann Graduate School of Biology and Medicine, University of Freiburg, Albertstr. 19A, 79104 Freiburg, Germany; 3 Faculty of Biology, University of Freiburg, Schänzlestr. 1, 79104 Freiburg, Germany

## Abstract

Here, we applied optoRAF, an optogenetic tool for light-controlled clustering and activation of RAF proteins that mimics the natural occurring RAS-mediated dimerization. This versatile tool allows studying the effect on BRAF and CRAF homodimer- as well as heterodimer-induced RAF signaling. Vemurafenib and dabrafenib are two clinically approved inhibitors for BRAF that efficiently suppress the kinase activity of oncogenic BRAF (V600E). However in wild-type BRAF expressing cells, BRAF inhibitors can exert paradoxical activation of wild-type CRAF. Using optoRAF, vemurafenib was identified as paradoxical activator of BRAF and CRAF homo- and heterodimers. Dabrafenib enhanced activity of light-stimulated CRAF at low dose and inhibited CRAF signaling at high dose. Moreover, dabrafenib increased the protein level of CRAF proteins but not of BRAF proteins. Increased CRAF levels correlate with elevated RAF signaling in a dabrafenib-dependent manner, independent of light activation.

Members of the RAF family of serine/threonine protein kinases play a central role in the mitogen-activated protein kinase (MAPK) pathway that includes the three-tiered protein kinase cascade RAF-MEK-ERK regulating cell proliferation, differentiation and cell survival^1^. Binding of a growth factor to its respective receptor tyrosine kinase stimulates the small GTPase RAS, forming plasma membrane nanoclusters^2^. Active RAS recruits cytosolic inactive RAF to the membrane and induces RAF dimerization^3^. Subsequent phosphorylation of activating sites in RAF leads to its full activation^4^,^5^. In contrast to ARAF and CRAF, mutants of BRAF have frequently been found in human tumors including metastatic melanoma and papillary thyroid carcinoma^1^,^6^. Most commonly, the substitution of Val 600 to Glu renders BRAF constitutively active in its monomeric form^4^. Additionally, BRAF mutants impaired in their kinase activity heterodimerize with wild-type CRAF to activate the MAPK pathway in an uncontrolled manner and drive tumor progression through CRAF^7^,^8^. ATP-competitive inhibitors such as vemurafenib and dabrafenib are approved for treatment of BRAF(V600E) positive metastatic melanoma and efficiently suppress RAF signaling^9^,^10^. However in cells expressing wild-type BRAF, RAF inhibitors can induce paradoxical activation of ERK^8^,^11^,^12^,^13^. Paradoxical activation of RAF signaling correlates with the appearance of keratoacanthomas and cutaneous squamous cell carcinoma as well as malignant melanocytic tumors and chronic lymphocytic leukemia^14^.

Optogenetic approaches have been developed for several signaling proteins including protein kinases enabling spatiotemporal control of their activity and uncoupling them from natural activators^15^,^16^. OptoCRAF, based on CRAF fused to the N-terminal photolyase homology region (PHR) of the *Arabidopsis thaliana* photosensor cryptochrome 2 (CRY2) was the first engineered light-controllable protein kinase^17^,^18^. Here, we extended the tool box for the optogenetic control of RAF for studying BRAF and CRAF homodimer- and heterodimer-dependent activation as well as paradoxical activation of RAF signaling. We applied this optoRAF system to characterize RAF inhibitors approved for treatment of human cancer.

## Results

### Design of optoRAF systems

**Figure 1:**
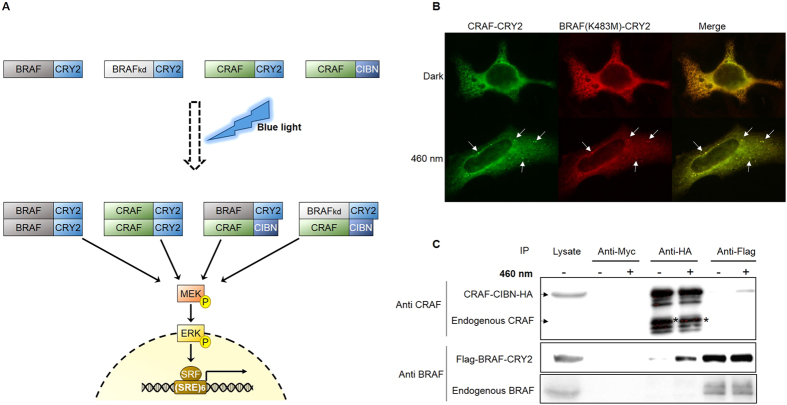
Design of the optoRAF systems. (**A**) Scheme of the RAF fusion proteins used to build up the optoRAF system for light-dependent activation of the MAPK pathway. (**B**) HeLa cells co-expressing CRAF-CRY2 and BRAF(K483M)-CRY2 were exposed to blue light of 460 nm or left in the dark before immunostaining was performed using Alexa Fluor 488 and Alexa Fluor 594. Blue-light induced co-clustering of CRAF-CRY2 and BRAF(K483M)-CRY2 is indicated by white arrows. (**C**) Lysate of HEK293T cells co-expressing Flag-BRAF-CRY2 and CRAF-CIBN-HA were incubated with different magnetic beads (anti-Myc, anti-HA, anti-Flag) under 460 nm light (+) or in darkness (−). The bound proteins were analyzed by immunoblotting with anti CRAF (CRAF) and anti BRAF (BRAF) antibodies. Signals derived from degraded overexpressed CRAF-CIBN-HA are marked by asterisks (*).

The light-based RAF systems consist of one of the human RAF isoforms BRAF or CRAF fused to the PHR domain of CRY2 (Fig. 1A, upper part). Flavin adenine dinucleotide (FAD) non-covalently linked to CRY2 serves as chromophore inducing CRY2 oligomerization upon exposure to blue light (460 nm) within seconds; oligomers monomerize again within minutes in the dark^19^,^20^. Thereby, BRAF or CRAF fused to the PHR-CRY2 domain (abbreviated with CRY2 hereafter) oligomerize likewise stimulating RAF signaling that can be monitored by phosphorylation and activation of the downstream protein kinases MAPK kinase/ERK kinase (MEK) and extracellular-regulated protein kinase (ERK) and ERK-dependent reporter gene expression (Fig. 1A, lower part). Thus, light-dependent interaction of CRY2 mimics RAS-mediated RAF dimer formation occurring under physiological conditions. To mimic RAF heteromerization, BRAF-CRY2 and CRAF fused to a truncated version of the CRY2 binding partner CIB1 (CIBN, residues 1–170)^19^ were co-expressed (Fig. 1A). CIBN on its own is unable to dimerize. The kinase-defective mutant BRAF(K483M) fused to CRY2 in combination with CRAF-CIBN was used to mimic the paradoxical activation of wild-type CRAF mediated by kinase-impaired mutants of BRAF.

Typically, CRY2 fusion proteins overexpressed in cells cluster upon exposure to blue light^20^. Consistently, immunofluorescence experiments indicated that CRAF and BRAF proteins fused to CRY2 cluster and co-localize in cells exposed to blue light as shown here for CRAF-CRY2 and BRAF(K483M)-CRY2 co-expressed in HeLa cells (Fig. 1B). To further verify light-dependent interaction between RAF-CRY2 and RAF-CIBN fusion proteins, we lysed HEK293T cells overexpressing HA-tagged CRAF-CIBN and FLAG-tagged BRAF-CRY2 and exerted co-immunoprecipitation assays. These assays were performed under continuous exposure to blue light to promote oligomerization and to prevent monomerization by dark reversion. BRAF-CRY2 co-immunoprecipitated with CRAF-CIBN and vice versa validating light-dependent interaction of RAF-CRY2 fusion proteins (Fig. 1C). Endogenous CRAF and BRAF could not be detected in these co-immunoprecipitation assays. However, expression of CRAF-CRY2 and BRAF-CRY2 were ten times and six times higher than their respective endogenous proteins (Supplementary Fig. S1).

**Figure 2:**
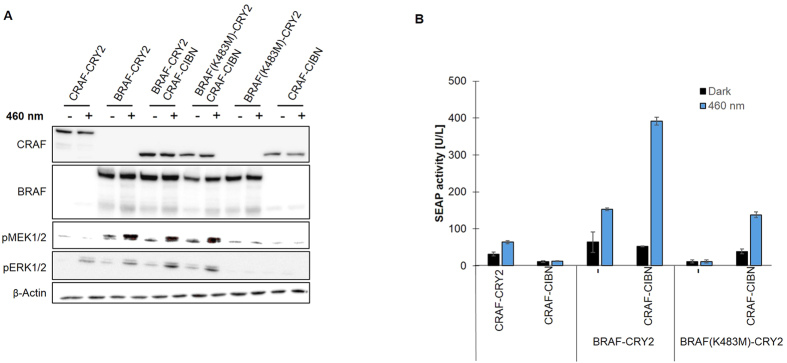
OptoRAF systems activate MAPK pathway (**A**) HEK293T cells were transfected with the plasmids as indicated and incubated for about 50 h in darkness (−) or illuminated for 5 min with light of 460 nm (+) before lysis of the cells. Cell lysates were immunoblotted with the respective antibodies to detect CRAF and BRAF and to analyze MEK and ERK activation by phospho-specific antibodies (pMEK1/2 and pERK1/2, respectively). β-actin was used as loading control. For quantification of the pERK1/2 levels see Supplementary Fig. S3. (**B**) HEK293T cells expressing the RAF constructs as indicated were incubated for 24 h and subsequently incubated for further 24 h in darkness (black columns) or exposed to continuous blue light (blue columns). SEAP activity was determined from the supernatants collected.

After confirming light-induced interaction of RAF-CRY2 and RAF-CIBN fusion proteins, we studied the ability of the optoRAF systems to stimulate RAF signaling. Stimulation of RAF activity was monitored by phosphorylation of MEK and ERK (Fig. 2A). Additionally, optoRAF-induced signaling was analyzed by reporter assays that monitor ERK activity by serum response element (SRE)-dependent expression of the secreted alkaline phosphatase (SEAP) (Fig. 2B)^18^. Both, BRAF-CRY2 and CRAF-CRY2 expressed in HEK293T cells activated the MAPK kinase cascade upon exposure to light of 460 nm as monitored by phosphorylation of MEK and ERK and increased reporter gene expression (Fig. 2A,B). The basal phosphorylation of MEK and ERK caused by BRAF-CRY2 was stronger compared to CRAF-CRY2, which may partially depend on a higher basal activity level of BRAF compared to CRAF. Co-expression of BRAF-CRY2 with CRAF-CIBN resulted in a pronounced light-dependent activation compared to BRAF-CRY2 alone (Fig. 2A,B). This indicates the higher activity of BRAF/CRAF heterodimers over BRAF or CRAF homodimers^21^. Co-expression of BRAF(K483M)-CRY2 and CRAF-CIBN mimicked paradoxical activation of CRAF by BRAF mutants with impaired kinase activity leading to substantial MEK/ERK phosphorylation and reporter gene activity (Fig. 2A,B)^18^. Unaccompanied expression of BRAF(K483M)-CRY2 and CRAF-CIBN served as control for basal activity of this pathway. As shown for BRAF-CRY2 rising light intensities correlated with increased BRAF signaling and reached an activity comparable to 10 ng/ml EGF in respect to ERK phosphorylation (Supplementary Fig. S2).

### Effect of inhibitors on BRAF and CRAF homodimer-dependent activation

Next we tested whether the optoRAF systems are applicable to characterize inhibitors of RAF kinase activity. Vemurafenib and dabrafenib are small molecule type-I class inhibitors that bind with the protein kinase in its active conformation. By interacting with the ATP-binding site they function as reversible ATP-competitive inhibitors. Vemurafenib and dabrafenib inhibit most efficiently the oncogenic mutant BRAF(V600E) with half maximal inhibitory concentrations in biochemical assays at IC_50_ = 31 nM and dabrafenib IC_50_ = 0.8 nM, respectively. However, they also inhibit CRAF and BRAF wild type protein at slightly higher concentrations. Vemurafenib and dabrafenib are approved for treatment of BRAF(V600E) positive metastatic melanoma^10^. GW5057 has been described as a CRAF selective kinase inhibitor^22^. Trametinib is an reversible allosteric inhibitor for MEK1 and MEK2 and in combination with dabrafenib approved for advanced melanoma^14^.

**Figure 3:**
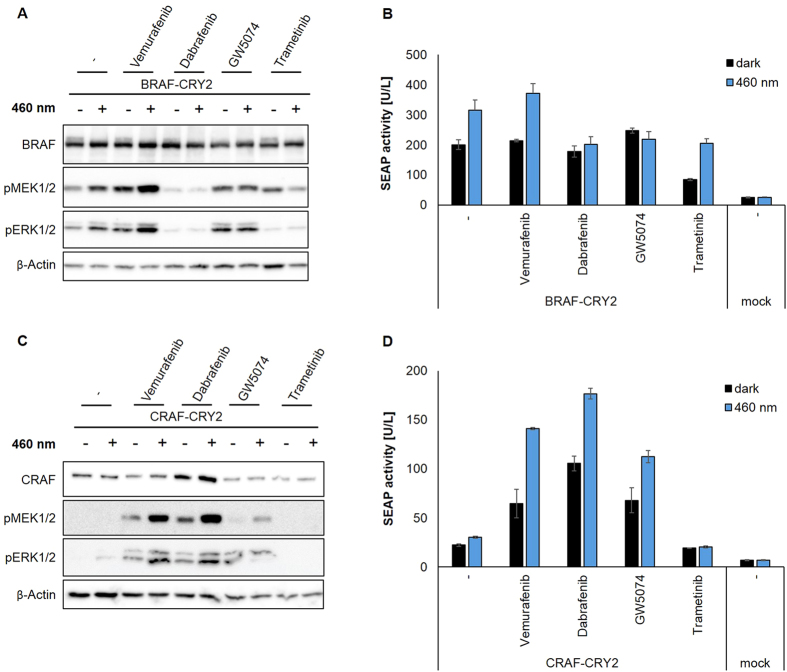
Effect of kinase inhibitors on light-induced BRAF and CRAF signaling. (**A**,**C**) HEK 293T cells expressing BRAF-CRY2 (**A**) or CRAF-CRY2 (**C**) were treated with the kinase inhibitors indicated for 26 h prior to further incubation in darkness (−) or illumination with blue light (+) for 5 min. Cell lysates were immunoblotted to detect BRAF, CRAF, pMEK and pERK as indicated. (**B**,**D**) SEAP activity was determined from supernatants of cells expressing BRAF-CRY2 (**B**) and CRAF-CRY2 (**D**) and treated with inhibitors as indicated for 2 h before further incubation in darkness (dark columns) or exposure to blue light (blue columns) for 24 h. Inhibitor concentrations used: dabrafenib 10 μM, vemurafenib 3 μM, GW5074 5 μM, trametinib 0.05 μM.

In a first step, we evaluated the effect of these inhibitors using our optogenetic-based systems for homodimer-dependent BRAF or CRAF activation. In cells expressing BRAF-CRY2, vemurafenib increased both basal and BRAF-CRY2 stimulated MEK and ERK phosphorylation (Fig. 3A). This increase is in line with previous observations that ATP-competitive inhibitors can promote activation of the MAPK kinase cascade^8^,^11^,^12^,^13^. Treatment with GW5074 also resulted in an increased MEK and ERK phosphorylation in darkness, however, it inhibited further activation of BRAF-CRY2 by light exposure. In contrast, dabrafenib did not increase basal activity of MEK and ERK and efficiently suppressed light-dependent activation of BRAF. Finally, the MEK inhibitor trametinib prevented basal and light-induced RAF signaling as shown by a low ERK phosphorylation level. It should be mentioned that trametinib did not prevent activating phosphorylation events on MEK, however, inhibited MEK-dependent substrate phosphorylation on ERK. In general, the SRE-dependent reporter assays confirmed the data obtained by MEK and ERK phosphorylation. This indicates that inhibitor effects on MEK/ERK phosphorylation measured after 5 min of light exposure correlates with reporter activity measured after 24 hours of light exposure (Fig. 3B). The only exception was observed for cells expressing BRAF-CRY2 and treated with trametinib. In this case the inhibitor was able to repress the light-induced short term activation monitored by ERK phosphorylation. Long term activation monitored by the reporter gene activity was reduced compared to non-treated cells expressing BRAF-CRY2. However, a light-dependent increase in SEAP activity was detectable. Either the trametinib concentration applied was too low to repress long term effects of light-induced BRAF or positive feedback mechanisms were stimulated in this setup. This effect has to be analyzed in further studies.

In cells expressing CRAF-CRY2, vemurafenib acts similar as in BRAF-CRY2 expressing cells. In both cases phosphorylation of MEK and ERK was increased (Fig. 3C, compared with Fig. 3A). However, treatment with dabrafenib had a different impact on CRAF-CRY2 expressing cells. In this case, dabrafenib did not inhibit but increased basal phosphorylation of MEK and ERK and further increased CRAF signaling induced by light exposure (Fig. 3C). Interestingly, dabrafenib seems to significantly enhance the protein levels of CRAF-CRY2. Treatment of CRAF-CRY2 expressing cells with GW5074 slightly increased MEK and ERK phosphorylation in dark and upon light exposure. Trametinib inhibited CRAF signaling. The SEAP reporter assays confirmed the effects of these inhibitors on MEK/ERK phosphorylation in CRAF-CRY2 expressing cells (Fig. 3D). All three RAF inhibitors tested clearly increased the basal reporter activity in CRAF-CRY2 expressing cells, whereas these inhibitors did not further increase the already elevated basal reporter activity in BRAF-CRY2 expressing cells.

Hence, vemurafenib and dabrafenib exert different effects in BRAF-CRY2 and CRAF-CRY2 expressing cells. In case of light-induced BRAF, vemurafenib enhanced and dabrafenib inhibited the RAF kinase cascade activity. Concerning light-induced CRAF, vemurafenib and dabrafenib, both elevated RAF-dependent signaling.

### Effect of inhibitors on BRAF and CRAF heterodimer-dependent activation

**Figure 4:**
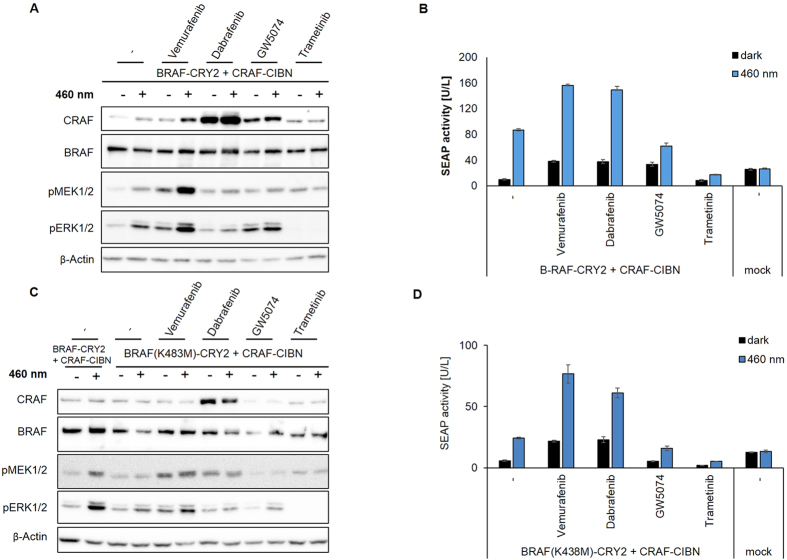
Effect of kinase inhibitors on light-induced BRAF/CRAF heterodimer signaling. (**A**,**C**) HEK293T cells co-expressing BRAF-CRY2 and CRAF-CIBN (**A**) or BRAF(K483)-CRY2 and CRAF-CIBN (**C**) were treated with inhibitors prior to further incubation in darkness (−) or illumination for 5 min with blue light (+). Analyses performed as in Fig.3 A/C. (**B**,**D**) SEAP activity of cells co-expressing BRAF-CRY2 and CRAF-CIBN (**B**) or BRAF(K483)-CRY2 and CRAF-CIBN (**D**) treated with inhibitors and further incubated in darkness or exposed to blue light. Analyses performed as in Fig. 3 B/D.

Besides homodimerization of RAF, BRAF/CRAF heteromerization is a physiological relevant mechanism to activate RAF signaling. We mimicked this mechanism by co-expressing BRAF-CRY2 and CRAF-CIBN. Blue light exposure is expected to result in active BRAF-CRY2/CRAF-CIBN heterodimers and active BRAF-CRY2 oligomers. Light stimulation of cells co-expressing these constructs displayed increased phosphorylation of MEK and ERK in line with enhanced SEAP reporter gene expression (Fig. 4A,B). In respect to vemurafenib, BRAF-CRY2 and CRAF-CIBN co-expression showed increased basal and light-induced phosphorylation of MEK and ERK correlating with elevated reporter gene expression similar to the results obtained with BRAF and CRAF homodimers (Fig. 4A,C, compared with Fig. 3A,C). In contrast, dabrafenib induced a slight paradoxical MEK/ERK activation in BRAF-CRY2/CRAF-CIBN co-expressing cells in dark and suppressed the light-dependent phosphorylation of MEK and ERK (Fig. 4A). Interestingly, under these conditions the dabrafenib-induced long term effect measured by SEAP activity was uncoupled from the MEK/ERK phosphorylation level and reached highly elevated levels (Fig. 4B). As observed above (Fig. 3C), dabrafenib treatment correlated with a higher level of the CRAF fusion protein, an effect that was not observed in respect to the level of BRAF proteins (Fig. 4A,B). In contrast to CRAF-CRY2 expressing cells (Fig. 3C), vemurafenib and dabrafenib also slightly elevated the level of CRAF-CIBN in cells co-expressing BRAF-CRY2 (Fig. 4A). However, also in this case, the expression level of BRAF-CRY2 is not affected. GW5074-increased basal phosphorylation of MEK and ERK in dark and prevented light-dependent RAF activation in BRAF-CRY2/CRAF-CIBN expressing cells (Fig. 4A). Increased basal activity could also be observed in the SEAP reporter assay, however, light-induction was slightly decreased compared to the non-inhibitor treated control (Fig. 4B). Trametinib inhibited both basal and light-induced BRAF/CRAF-dependent ERK phosphorylation and ERK signaling. Blue light stimulation of BRAF(K483M)-CRY2/C-RAF-CIBN only enables the transactivation of CRAF by kinase-defective BRAF, as BRAF(K483M)-CRY2 oligomers are expected and have been shown to be inactive^18^. This is reflected by general lower activation levels of MEK and ERK and SEAP reporter activity. However, overall effects obtained by inhibitor treatment of BRAF(K483M)-CRY2/C-RAF-CIBN expressing cells were similar to BRAF-CRY2/CRAF-CIBN co-expression (Fig. 4C,D).

### Concentration-dependent effects of dabrafenib on CRAF signaling

**Figure 5:**
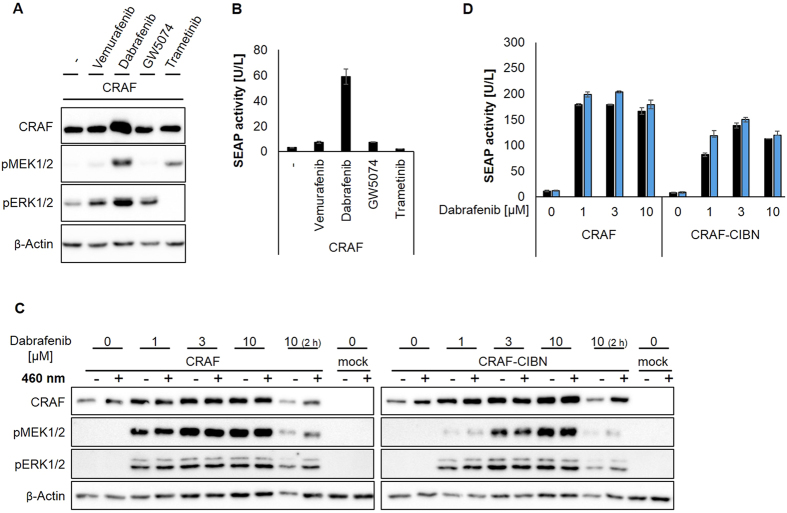
Effects of RAF inhibitors on light-insensitive CRAF. (**A**,**B**) HEK293T cells expressing CRAF were treated with the inhibitors as indicated for about 24 h and analyzed by immunoblotting (**A**) and SEAP reporter assay (**B**) as described in Fig. 3 (**C**) HEK293T cells were transfected with CRAF, CRAF-CIBN or empty vector control (mock) and treated with increasing concentrations of dabrafenib for 26 h before the cells were incubated in darkness (−) or exposed to blue light for 5 min (+). **(D)** For SEAP reporter assays HEK293T cells expressing CRAF or CRAF-CIBN were treated with increasing concentrations of dabrafenib for 2 h before they were further incubated in darkness (dark columns) or illuminated with blue light (blue columns) for 24 h. Analyzes by immunoblotting (**C**) and SEAP reporter assay (**D**) were performed as described in Fig. 3.

**Figure 6:**
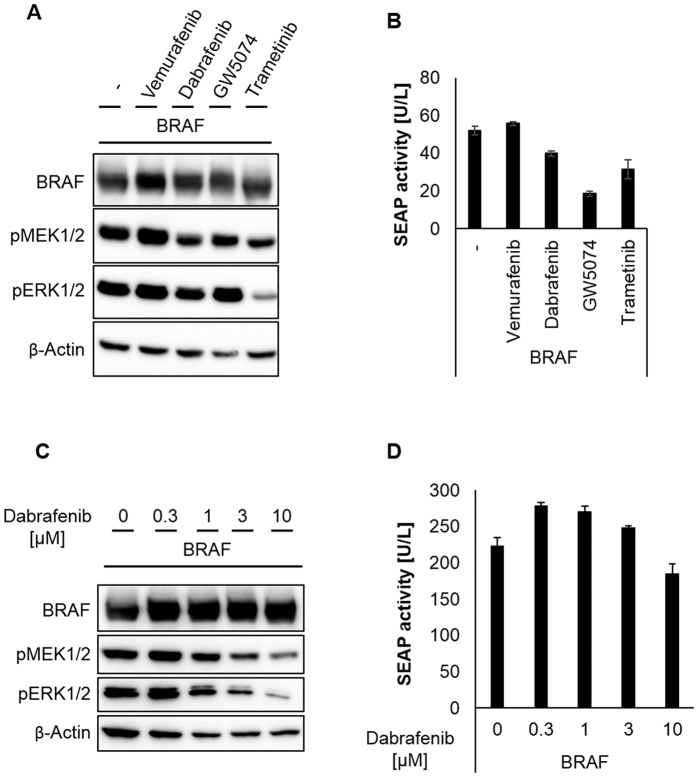
Effect of RAF inhibitors on BRAF. (**A**,**B**) HEK293T cells expressing BRAF were treated with the inhibitors as indicated for 26 h and analyzed by immunoblotting (**A**) and SEAP reporter assay (**B**) as described in Fig. 3 (**C,D**) HEK293T cells expressing BRAF were treated with increasing concentrations of dabrafenib before the cells were incubated in darkness or exposed to blue light as described in Fig. 3.

Dabrafenib elevated the protein levels of CRAF-CRY2 and CRAF-CIBN fusion proteins in cells, an effect independent of light exposure (Figs 3C, 4A,C). In addition, in case of CRAF-CRY2, dabrafenib treatment and hence elevated protein levels correlated with increased RAF signaling as monitored by MEK and ERK phosphorylation and reporter gene expression (Fig. 3C,D). To exclude influences of CRY2 and CIBN fused to CRAF we included non-fused wild-type CRAF in our study. CRAF overexpressing cells were treated with the above mentioned inhibitors. Dabrafenib-treatment resulted in a strong inducing effect and potently increased MEK and ERK phosphorylation and enhanced SRE-dependent reporter gene expression by 18-fold (Figs 5A and 4B). In contrast, vemurafenib and GW5074 only slightly induced ERK phosphorylation and SRE-dependent reporter gene expression (Fig. 5A,B). In case of wild-type BRAF, dabrafenib did not elevate but significantly decreased MEK and ERK phosphorylation and SRE-dependent reporter gene expression induced by overexpressed BRAF (Fig. 6A,B). While vemurafenib slightly induced paradoxical activation in BRAF expressing cells, GW5074 and trametinib reduced BRAF signaling (Fig. 6A,B).

**Figure 7:**
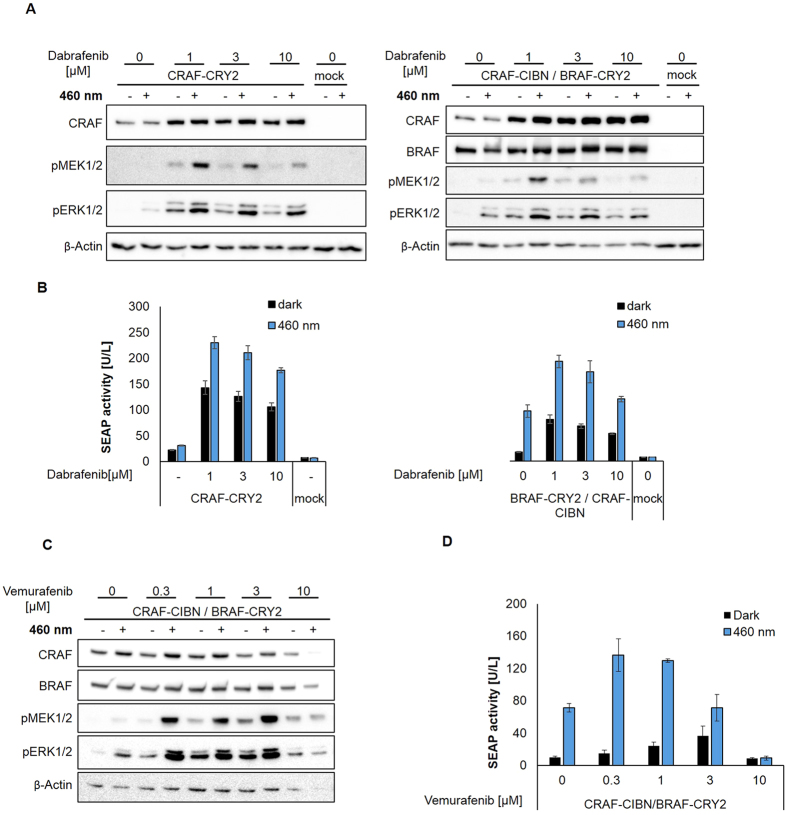
Effect of dabrafenib and vemurafenib on opto-RAF. (**A**) HEK 293T cells transfected with CRAF-CRY2 or CRAF-CIBN and BRAF-CRY2 were treated with increasing concentrations of dabrafenib for 26 h prior to incubation in darkness (−) or illumination with blue light (+) for 5 min. Cell lysates were immunoblotted to detect BRAF, CRAF, pMEK, pERK and β-actin as indicated. (**B**) SEAP activity was determined from supernatants of cells expressing CRAF-CRY2 or CRAF-CIBN and BRAF-CRY2 and treated with increasing concentrations of dabrafenib for 2 h before further incubation in darkness (dark columns) or exposure to blue light (blue columns) for 24 h. Supernatants of cells were used to determine SEAP activity. (**C**,**D**) HEK 293T cells co-transfected with CRAF-CIBN and BRAF-CRY2 were treated with increasing concentrations of vemurafenib and analyzed as described for dabrafenib in (**A**,**B**).

So far, in all experiments treatment with dabrafenib was performed for about 24 hours at a concentration of 10 μM. We next tested different concentrations of dabrafenib. Already at a concentration of 1 μM, dabrafenib elevated the protein level of the light-insensitive constructs CRAF and CRAF-CIBN, higher concentrations tested up to 10 μM further increased their protein level (Fig. 5C). Correlating with the increased protein level, phosphorylation of MEK and ERK increased. SEAP activity reached a maximum between 1 and 3 μM dabrafenib and a high level of SEAP activity was maintained with 10 μM dabrafenib (Fig. 5D). Treatment with 10 μM dabrafenib for two hours did not significantly increase CRAF and CRAF-CIBN expression but still led to slightly elevated MEK and ERK phosphorylation, indicating that the MAPK cascade activation by dabrafenib is not solely attributable to the CRAF protein level (Fig. 5C). However, the light-insensitive RAF proteins, CRAF and CRAF-CIBN, seem to be stabilized by dabrafenib treatment associated with enhanced stimulation of RAF signaling. The effect of dabrafenib on the protein level is specific for the isoform CRAF as the protein level of BRAF is not significantly affected (Fig. 6C). In addition, increasing concentrations of dabrafenib inhibited the signaling induced by overexpressed BRAF as monitored by the phosphorylation of MEK and ERK and SRE-dependent reporter gene expression (Fig. 6C,D). As is the case for CRAF and CRAF-CIBN, the protein level of the light responsive CRAF-CRY2 rose with 1 μM dabrafenib and only slightly increased with higher concentrations of dabrafenib (Fig. 7A). However, in contrast to the light-insensitive RAF proteins, light-stimulated CRAF-CRY2 signaling seems to reach its maximum at 1 μM dabrafenib and declined with higher concentrations (Fig. 7A). Correlating with the elevated expression level, CRAF-CRY2 showed an increase in MEK and ERK phosphorylation as well as SRE-dependent reporter gene expression in the presence of 1 μM dabrafenib, but remained unaffected by higher concentration of dabrafenib (Fig. 7A,B). The reverse effect between increasing concentration of dabrafenib and RAF signaling shown for light-stimulated CRAF-CRY2 is even more pronounced in the setup using the combination of BRAF-CRY2 and CRAF-CIBN (Fig. 7A,B). This indicates that light-induced CRAF dimerization by overexpressed CRAF-CRY2 or co-expressed BRAF-CRY2/CRAF-CIBN, in combination with small amounts of dabrafenib elevates both CRAF protein levels and downstream signaling. Higher concentrations of dabrafenib inhibited RAF signaling without further affecting the level of CRAF proteins. To further compare dabrafenib with vemurafenib, BRAF-CRY2/CRAF-CIBN expressing cells were treated with increasing concentration of vemurafenib (Fig. 7C,D). Increasing concentrations of vemurafenib up to 3 μM elevated phosphorylation of MEK and ERK in light-activated cells expressing BRAF-CRY2 and CRAF-CIBN. SRE-dependent gene expression reached a maximal stimulation by 0.3 μM vemurafenib and decreased with higher concentrations. In contrast to dabrafenib, vemurafenib at a concentration of 10 μM is cytotoxic as detectable by decreased levels of β-actin used as loading control.

## Discussion

Here, we applied the optoRAF system depending on light-controlled BRAF and CRAF proteins to characterize the clinically approved BRAF inhibitors vemurafenib and dabrafenib^9^. According to the effect of RAF inhibitors in the context of constitutively active RAS and wild-type RAF proteins^8^,^11^,^12^,^13^, vemurafenib paradoxically activated RAF signaling in BRAF-CRY2 and CRAF-CRY2 expressing cells already in dark. This effect was further enhanced by light exposure indicating that vemurafenib does not inhibit RAF dimer-induced signaling in our optoRAF setup. In the heterodimeric BRAF-CRY2/CRAF-CIBN system the effect of vemurafenib was even more pronounced in respect to stimulation of SRE-dependent gene expression. Interestingly, vemurafenib further activated signaling of BRAF(K483M)-CRY2/CRAF-CIBN expressing cells that mimics the paradoxical activation of CRAF by a kinase-impaired BRAF mutant. Thus, vemurafenib can still bind to kinase-dead BRAF and promotes its effect to transactivate wild-type CRAF. Vemurafenib led to a significant stimulation of homo- and heterodimeric RAF, indicating that basal signaling in starved HEK293T cells can promote paradoxical stimulation by RAF inhibitors.

In some aspects, dabrafenib behaved differently to vemurafenib. In BRAF-CRY2 expressing cells, dabrafenib was unable to induce paradoxical activation and inhibited light-stimulated phosphorylation of MEK and ERK as well as SEAP activity. In contrast, in CRAF-CRY2 expressing cells dabrafenib induced paradoxical activation of RAF signaling in dark which was even more pronounced upon light exposure. This is consistent with the observation that paradoxical activation by dabrafenib depends exclusively on CRAF but not BRAF or ARAF^23^. Light-dependent homodimerization of CRAF-CRY2 or heterodimerization of CRAF-CIBN with BRAF-CRY2 enabled dabrafenib to induce paradoxical CRAF signaling at low concentrations. At higher concentrations dabrafenib inhibited CRAF signaling. This is in agreement with the current models for RAF activation^13^,^5^,^24^ proposing that at unsaturated concentrations, inhibitors bind to one protomer enabling transactivation or relieve of autoinhibition of the second inhibitor-free protomer, whereas, at higher concentrations inhibitors bind to both protomers suppressing CRAF signaling. This behavior could not be observed for vemurafenib under the conditions used in this study.

In cells expressing light-controlled CRAF-CRY2, dabrafenib significantly induced basal signaling in the dark. Consistently, dabrafenib activated non-fused wild-type CRAF and light-insensitive CRAF-CIBN, whereas the activity of BRAF with or without CRY2 fusion was inhibited. Interestingly, even at a concentration of 10 μM, dabrafenib induced a robust stimulation of CRAF signaling independent of light- or RAS-mediated CRAF dimer formation in serum-starved HEK293T cells expressing wild-type CRAF or the light-insensitive CRAF-CIBN protein. Most ATP-competitive inhibitors induce dimer formation of its targets, although in case of RAF this effect can be promoted by activated RAS. Thereby, inhibitor-induced dimers stabilize an active, rigid closed conformation of the kinase domain^25^. In this study, cells were treated with RAF inhibitors for 24 h, in contrast to previous studies treating cells with dabrafenib for a shorter time period (1 h) to analyze effects on MEK and ERK phosphorylation^23^. Thus, prolonged treatment of cells with dabrafenib seems to stabilize the CRAF protein and thereby elevates its protein level, probably by accumulating CRAF dimers. Dabrafenib-increased CRAF protein levels have different outputs. In light-insensitive systems an increasing dose of dabrafenib correlates with an elevated CRAF protein level and CRAF signaling. Contrary, in case of light-activated CRAF an increasing dose of inhibitor first enhances and then reduces CRAF signaling despite of increasing CRAF protein levels. The effects of dabrafenib are specific for CRAF and did not occur with the other inhibitors tested.

Taken together, we applied a system based on light-regulated BRAF and CRAF to characterize RAF inhibitors. In contrast to other available cell-based systems, optoRAF allows activation of the RAF-MEK-ERK protein kinase cascade uncoupled from upstream activators and is a versatile tool to study inhibitors on RAF signaling in an isoform specific and homo- and heteromer-dependent manner. Recently developed paradox breakers avoiding paradoxical activation of RAF are attractive clinical candidates that can be further characterized by the optoRAF systems^26^,^27^.

## Methods

### Cloning procedures

The plasmids pRG300 and pGR302 for expression of BRAF and BRAF-CRY2, respectively, were generated by introducing PCR amplified DNA sequences for wild-type BRAF^28^ (Table S1) into the pGR59 mammalian expression vector published by Wend *et al*.^18^. All further plasmids were published elsewhere^18^.

### Antibodies

For Western-blotting, monoclonal Raf-B (F-7) antibody (Santa Cruz Biotechnology, Dallas,TX, cat no. sc-5284) was used at 1000-fold dilution. Polyclonal c-Raf (Cell Signaling Technology, Danvers, MA, cat no. 9422) and Phospho-p44/42 MAPK (Erk1/2) (Thr202/Tyr204) (Cell Signaling Technology, cat no. 4370) antibodies were used at a 2000-fold dilution. Phospho-MEK1/2 (S217/221) (Cell Signaling Technology, cat no.9154S) as well as sheep anti-mouse IgG-HRP (GE Healthcare Europe GmbH, Freiburg, Germany, cat. no. NA931) were applied as a 5000-fold dilution and goat anti-rabbit IgG-HRP (Santa Cruz Biotechnology, Dallas,TX, cat no. sc-2004) was used at a 10000-fold dilution in phosphate buffered saline (PBS) containing 0.05% (v/v) Tween-20 and 4% (w/v) milk powder.

For immunostaining, monoclonal Anti-Flag M2 (Sigma-Aldrich, cat. no. F3165) and monoclonal anti-C-RAF antibody (Cell signaling, cat. no. 9422) were used as primary antibodies at 400-fold dilution, and Alexa Fluor 594 anti-mouse antibody (Life Technologies, cat. no. A-11062) and Alexa Fluor 488 anti-rabbit antibody (Life Technologies, cat. no. A-11008) were used as secondary antibodies at 750- or 500–fold dilution, respectively.

For immunoprecipitation experiment, magnetic beads coated with anti-HA (Thermo scientific cat. no. 88836), anti-Flag (Sigma-Aldrich cat. no. M8823) and anti Myc (Thermo Fisher Scientific, cat. no. 88824) were used.

### Cell Culture

Human embryonic kidney HEK-293T cells were maintained in Dulbecco’s modified Eagle medium (DMEM, PAN Biotech GmbH, Aidenbach, Germany, cat. No. P03-0710) supplemented with 10% fetal calf serum (FCS, PAN Biotech GmbH, cat. no. 1502, batch P123002) and 1% (v/v) penicillin/streptomycin (PAN Biotech GmbH, cat. no. P06-07100) at 37 °C in a humidified atmosphere containing 5% CO_2_. Transfections were conducted by seeding 75 000 cells per well in 24-well plates. After 24 h, 0.7 μg plasmid DNA and 2.2 μL of PEI, (linear, MW = 25 kDa, Polysciences Europe GmbH, Eppelheim, Germany, cat. no. 23966-2) (1 mg/mL) per well were assembled in 40 μl cell culture volume of OptiMEM (Life technologies, cat. no. 22600-134), incubated at room temperature for 20 min and then added dropwise to the cells. eGFP cloned in the vector pEF6/V5-His-TOPO was used for mock transfections. 24 h post transfection, the cell culture medium was exchanged with starvation medium (DMEM, 1% (v/v) penicillin/streptomycin). In co-expression experiments, single construct controls were co-transfected with mock plasmid and the plasmids were applied in a ratio of 1 : 2 : 0.5 (w/w), for B-RAF, C-RAF and the SEAP reporter plasmid, respectively.

### Kinase inhibitors

Inhibitors were applied to the transfected cells 2 h prior illumination for 24 h. All inhibitors are from Selleckchem, Houston, TX and were prepared as stock solutions in dimethyl sulfoxide (DMSO), stored at −80 °C. Unless indicated otherwise, the final concentrations of the inhibitors used were 10 μM for dabrafenib (cat. no. S2807), 3 μM for vemurafenib (cat. no. S1267), 5 μM for GW5074 (cat. no. S2872) and 0.05 μM for trametinib (cat.no. S2673).

### Cell Lysis

For cell lysis, cell culture medium was removed and 100 μL of ice-cold lysis buffer (20 mM Tris/HCl, 100 mM NaCl, 1 mM EDTA, 0.5% (v/v) Triton X-100, 0.1% (w/v) SDS, pH 7.5) supplemented with protease (complete protease inhibitor cocktail, Roche, Basel, CH, cat. No. 04693116001) and phosphatase inhibitors (1 mM sodium orthovanadate, 10 mM sodium pyrophosphate, 50 mM sodium fluoride, 10 mM β-glycerophosphate) were added per 24 well. After 10 min incubation on ice, the cells were scraped off, and lysates were sonicated 4 times 30 sec each. Afterwards, lysates were centrifuged at 10,000 × *g* and 4 °C for 4 minutes, supernatants of 24-well triplicates were pooled and transferred to fresh tubes, mixed with 5× SDS sample buffer and boiled at 95 °C for 5 min.

### Western Blotting

Proteins were separated on 9% (w/v) sodium dodecyl sulfate polyacrylamide gel electrophoresis (SDS-PAGE) gels at 90 V following transfer to PVDF membranes (Immobilon-P Membrane, Merck KGaA, Darmstadt, Germany, cat. no. IPVH00010) at 350 mA per blot for 1 h. Membranes were blocked in PBST (PBS containing 0.05% (v/v) Tween-20) supplemented with 8% (w/v) milk powder (Carl Roth GmbH & Co.KG, Karlsruhe, Germany, cat. no. T145.3) for 1 h at room temperature. Primary antibody incubation was conducted at 4 °C overnight in PBST containing 4% (w/v) milk powder. After three washing steps with PBST, blots were incubated with the corresponding secondary antibody coupled to horseradish peroxidase (HRP) at room temperature for 1 h. After washing steps with PBST, chemiluminescence was detected by incubation in ECL I (1.25 mM luminol, 0.2 mM coumaric acid, 0.1 M Tris/HCl, pH 8.5) and ECL II (0.1 M Tris/-HCl, 0.01% H_2_O_2_, pH 8.5) reagents in a 1:1 ratio and using the LAS-4000 mini image analyzer (GE Healthcare Europe GmbH, Freiburg, Germany, cat. no. 28-9558-13). For detecting a second protein of interest on the same membrane, the membrane was incubated in 0.1% (w/v) sodium azide for 5 min, washed with H_2_O and PBST, and incubated over night with the respective primary antibody. Next steps were performed as described before. Band intensities for pERK were calculated using the program ImageJ and normalized using β-actin loading control as reference (Supplementary Fig. S3).

### Secreted Alkaline Phosphatase (SEAP) Assay

For SEAP assay analysis, cells were co-transfected with different RAF-constructs and the pERK2 inducible SEAP reporter (pGR53)^18^. as mentioned above. The cell culture supernatants were transferred to a round bottom 96 well-plate and heated at 65 °C for 30 min to inactivate endogenous phosphatases. After centrifugation at 1250 rpm for 1 min 80 μL samples were diluted in 100 μL 2× SEAP buffer (20 mM homoarginine, 1 mM MgCl_2_, 21% (v/v) diethanolamine, pH 9.8) and transferred into a flat bottom 96 well-plate. 20 μL 120 mM p-nitrophenyl phosphate (pNPP) solution was added and the absorbance was measured immediately at 405 nm excitation wavelength for 1 h using a microplate reader (Multiscan GO, Thermo Scientific, Waltham, MA). The SEAP activity was determined as described previously^18^. Average values and standard deviations from triplicates were determined.

### Immunoprecipitation experiment

To verify the potential of BRAF-CRY2 and CRAF-CIBN to heteromerize upon blue light exposure, an immunoprecipitation assay was performed. The lysate of a confluent 10 cm petri dish of HEK293T cells expressing Flag-BRAF-CRY2 and CRAF-CIBN-HA was loaded on 15 μl of different magnetic beads. Anti-HA beads, and anti-Flag beads were used to catch CRAF-CIBN-HA and Flag-BRAF-CRY2 respectively, whereas anti-Myc beads were used as a negative control. Beads and lysate were incubated either in dark or under blue light (460 nm) at 4 °C for 2 h on a rotating wheal. Beads were washed twice with lysis buffer (50 mM Tris pH 7.5, 100 mM NaCl, 5 mM β-mercaptoethanol, 1% Triton X-100, protease inhibitor (complete protease inhibitor cocktail, Roche, Basel, CH, cat. No. 04693116001) and once with wash buffer (50 mM Tris pH 7.5, 100 mM NaCl, 5 mM β-mercaptoethanol, 0.1% Triton X-100, protease inhibitor), resuspended in 50 μl SDS loading buffer, boiled at 90 °C for 5 minutes and 10 μl were loaded on a SDS gel for immunoblot analysis of proteins content.

### Immunofluorescence

To demonstrate the oligomerization potential of the CRY2 fusion proteins within cells, an immunofluorescence staining was performed. HeLa cells were seeded on coverslips and transfected with desired plasmids. 24 h post transfection, cells were either kept in dark or exposed to blue light 5 μE m-2 s-1 for 10 minutes. Cells were washed in PBS and fixed in a 1:1 ice-cold methanol/acetone solution at −20 °C for 20 minutes, and rinsed with PBS. Samples were blocked in 5% (w/v) BSA in PBS at room temperature for 30 min and incubated with primary antibodies at room temperature for 1 h. After extensive washing steps in 0.05% (v/v) PBST, samples were incubated at room temperature for 1 h with secondary antibodies and washed in PBS-T. Finally coverslips were mounted on microscope slides with Mowiol (Sigma Aldrich cat. 81381) and dried at room temperature overnight. Samples were analyzed using Axiovert 200 inverted fluorescence microscope (Carl Zeiss Microscopy GmbH) equipped with LD-Achroplan 10×, 20× or 100× objectives and AxioCam digital fluorescent camera. Pictures were acquired using the AxioVision imaging software (Carl Zeiss Microscopy GmbH; Release 4.8.1).

### Cell Illumination

48 h post transfection, cells expressing CRY2- and CIBN-fusion proteins were illuminated with in self-built light boxes containing 460 nm light-emitting diodes with a radiation angle of 120° of which the light intensities were adjusted to 5 μE m-2 s-1 using a quantum sensor (LI-250A Light Meter, LI-COR, Lincoln, NE). For analysis of the long-term effect of RAF activation via the SEAP assay, cells were illuminated for 24 h before 150 μl of the supernatant were transferred to a separate well plate. Afterwards, the cells were incubated for 2 h under dark conditions, sufficient to reverse activation of the RAF proteins as demonstrated previously^18^. For Western blot analysis and determination of the short-term effect of RAF activity, the cells were then illuminated for 5 min under the described light conditions followed by cell lysis. To protect the dark control from light, all experiments took place under red safelight conditions emitted by LEDs (Osram LED DECO^®^ RGB).

## Additional Information

**How to cite this article**: Chatelle, C. V. *et al*. Optogenetically controlled RAF to characterize BRAF and CRAF protein kinase inhibitors. *Sci. Rep.* **6**, 23713; doi: 10.1038/srep23713 (2016).

## Supplementary Material

Supplementary Information
