# Supplementary material for: Optogenetically controlled RAF to characterize BRAF and CRAF protein kinase inhibitors
Source: Sci Rep. 2016 Mar 30;6:23713. doi: 10.1038/srep23713 (PMC4812324; doi:10.1038/srep23713)
Supplement: Supplementary Information [file srep23713-s1.pdf]

Supplementary Information

Optogenetically controlled RAF to characterize BRAF and CRAF protein kinase inhibitors

Claire V. Chatelle[1,3+] ,Désirée Hövermann[1,3+],Anne Müller[3], Hanna J. Wagner[1,2,3], Wilfried Weber[1,2,3] and Gerald Radziwill[1,3 *]

[1]BIOSS - Centre for Biological Signalling Studies, University of Freiburg, Schänzlestr. 18, 79104 Freiburg, Germany

[2]SGBM - Spemann Graduate School of Biology and Medicine, University of Freiburg, Albertstr. 19A, 79104 Freiburg, Germany

[3]Faculty of Biology, University of Freiburg, Schänzlestr. 1, 79104 Freiburg, Germany

[+] These authors contributed equally to this work.

* Correspondence: gerald.radziwill@bioss.uni-freiburg.de

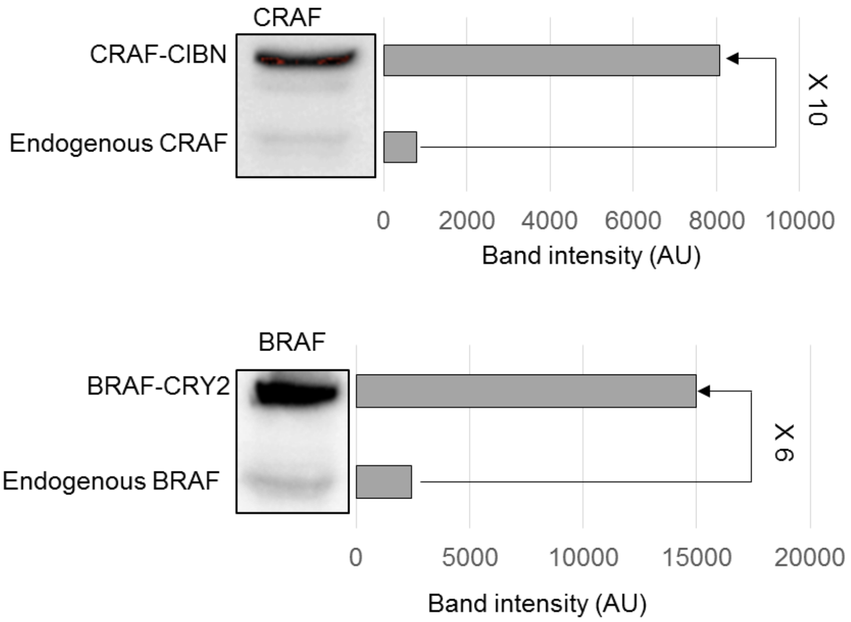

Figure S1: Expression levels of chimeric RAF proteins compared to endogenous RAF proteins. HEK293T cells coexpressing BRAF-CRY2 and CRAF-CIBN were lysed and immunoblotted with anti-CRAF and anti-BRAF antibodies. Band intensities were calculated using ImageJ and expression levels of the chimeric and endogenous RAF proteins were compared.

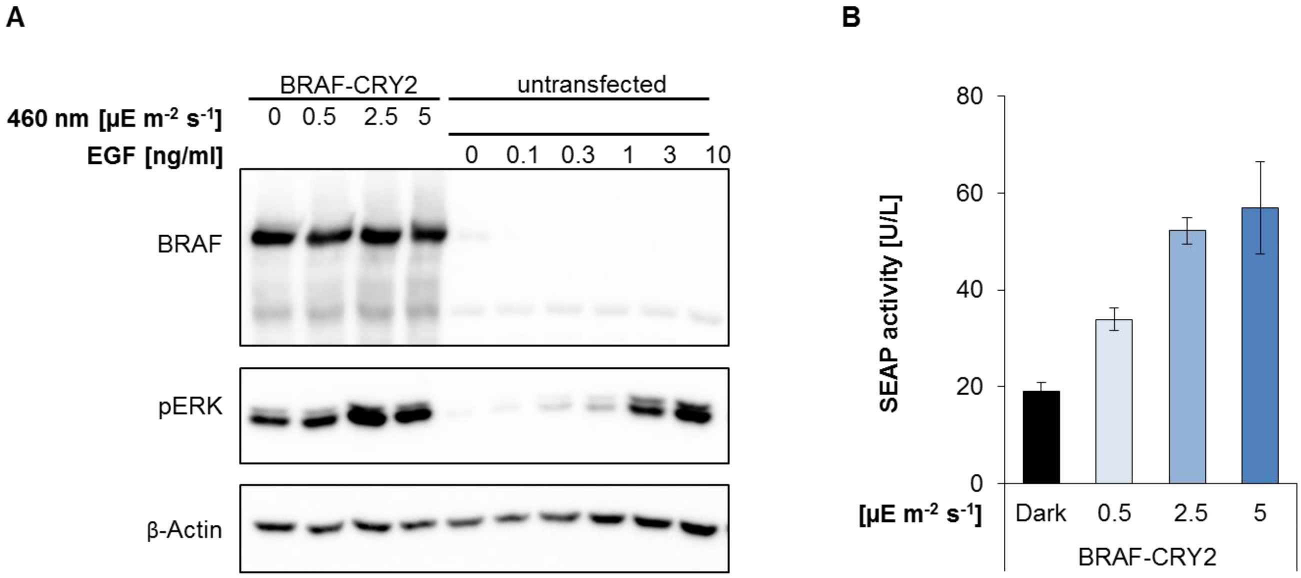

Figure S2. Light dose dependent stimulation of BRAF-CRY2. (A) HEK293T cells expressing BRAF-CRY2 were illuminated for 5 min with increasing intensity of light with 460 nm (left). Untransfected HEK 293T cells were incubated with increasing concentrations of EGF for 5 min. Cells were lysed and immunoblotted to detect BRAF and phospho-ERK (pERK), and β-actin as loading control. (B) Supernatant of BRAF-CRY expressing cells exposed to blue light with intensities as indicated were analyzed for SEAP activity.

Figure 2A

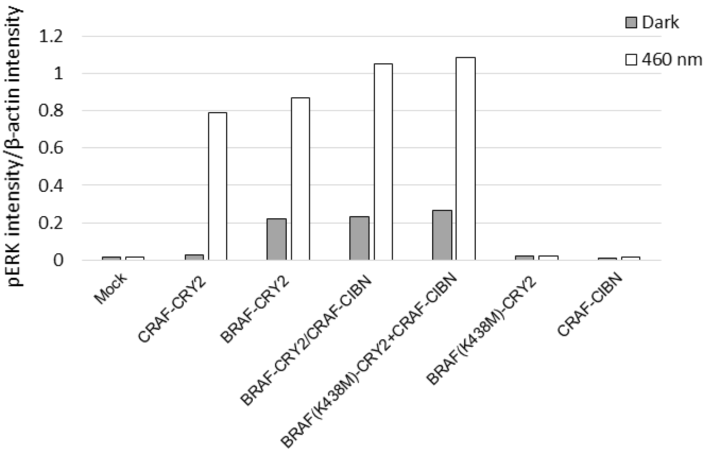

Figure 3

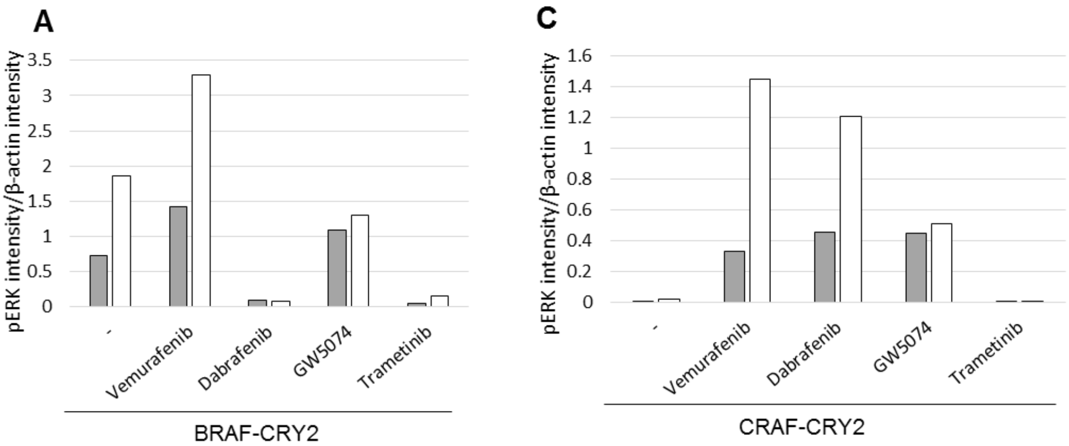

Figure 4

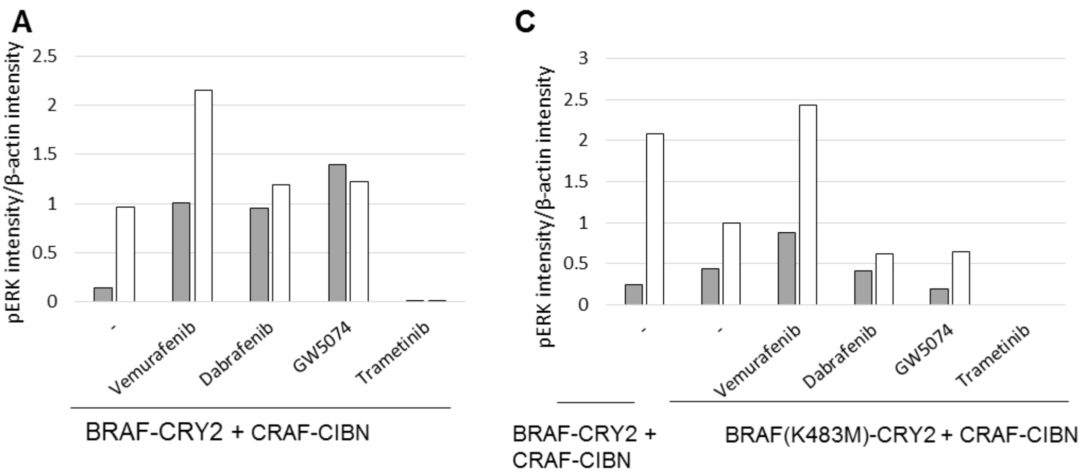

Figure 5

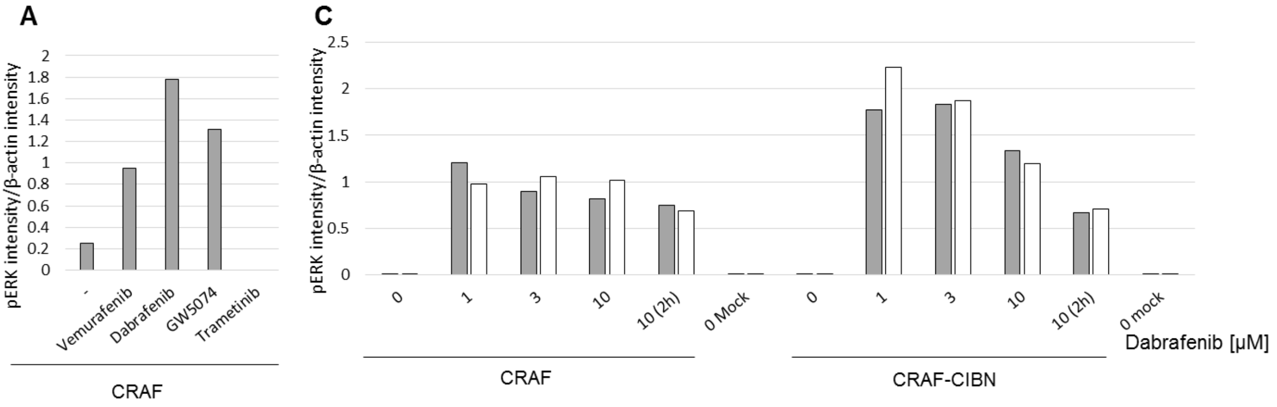

Figure 6

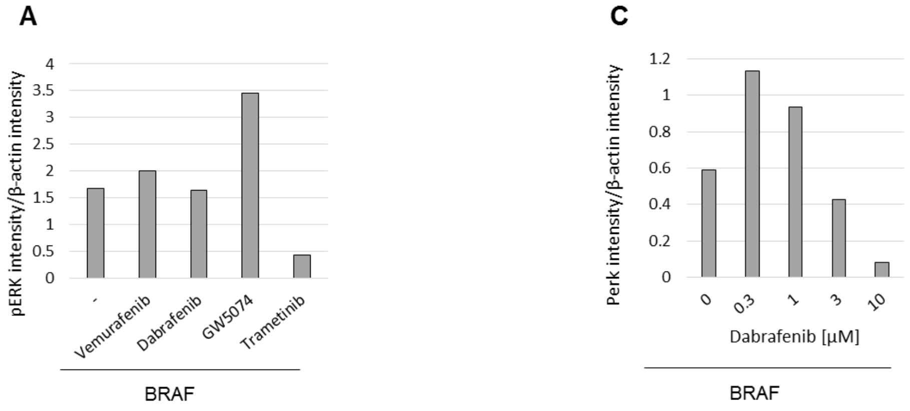

Figure 7

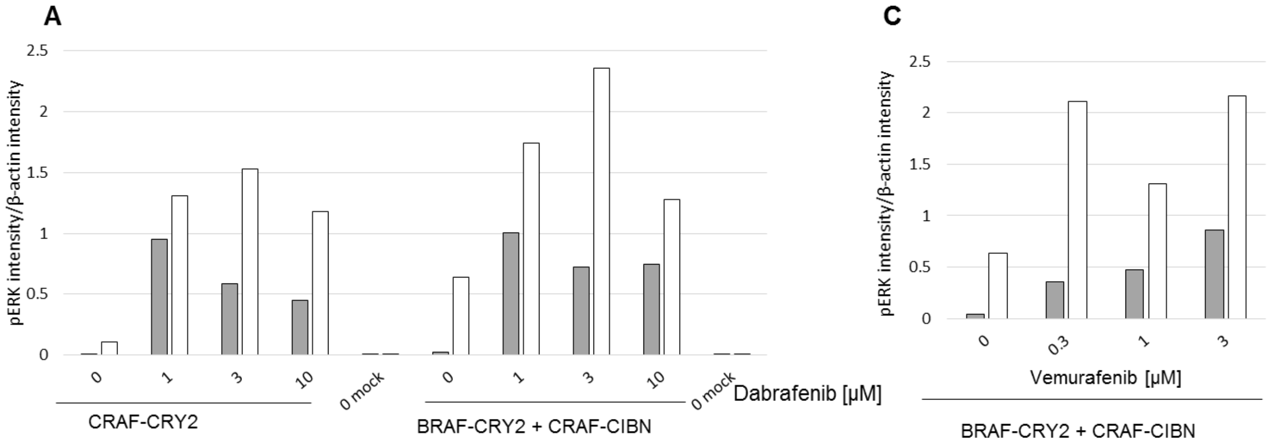

Figure S3. Quantification of pERKlevels. For every western blot analysis performed shown in this study pERK levels were quantified using the software ImageJ. Band intensities were calculated and normalized using β-actin loading control as reference. Grey lines and white lines present results obtained after dark and 460 nm light incubation, respectively.

Table 1: Primers designed and used for PCR amplification in this work. The annealing sequence is underlined.

Primer	Sequence (5' → 3')	Description
oGR307	GCTGGCTAGGTAAGCTTGGTACCGGCGCG**CCACCATGGACTACAAGGACGACGATGACAAGGGTGGTTCTGG**TATGGCGGCGCTGAGC	Forward primer for amplification of FLAG-*BRAF* and cloning of pGR300 and pGR302
oGR308	CTATAGTCTTTTTGTCCATCTTCATCATA**TGACCAGCACTACCAGCACTACCAGCACTACCAGCACTATC**GTGGACAGGAAACGCACC	Reverse primer for amplification of FLAG-*BRAF* and cloning of pGR302
oGR369	ATAGGCTTACCTTCGAACCGCGGGCCCTCTAGTTAGTGGACAGGAAACG	Reverse primer for amplification of FLAG-*BRAF* and cloning of pGR300
